# A multicenter prospective study on the management of hepatoblastoma in children: a report from the Chinese Children’s Cancer Group

**DOI:** 10.1007/s12519-023-00750-6

**Published:** 2023-09-28

**Authors:** Meng-Jie Tang, Xiao-Li Ma, Xiang-Ling He, Wei-Hua Pan, Xiao-Hong Zhang, Sha-Yi Jiang, Ju Gao, Fu Li, Wei Yao, Song Gu, Wei-Ling Zhang, Qiang Zhao, Shi-Hao Huang, Yong-Jun Fang, Wei Liu, Hui-Zhong Niu, Chun-Mei Wang, Li-Rong Sun, Hui Gao, Yun-Peng Dai, Shun-Gen Huang, Zhi-Yong Zhong, Xi-Ge Wang, Zhong-Rong Li, Liang-Chun Yang, Ye-Ming Wu, Huan-Min Wang, Xin Sun, Xiao-Jun Yuan

**Affiliations:** 1 Department of Pediatric Hematology/Oncology, Xinhua Hospital Affiliated to Shanghai Jiao Tong University School of Medicine, No. 1665, Kongjiang Road, Yangpu District, Shanghai, 200092 China; 2 Medical Oncology Department, Pediatric Oncology Center, Beijing Children’s Hospital, Capital Medical University, National Center for Children’s Health, Beijing, 100045 China; 3 Department of Hematology/Oncology, Children’s Medical Center, Hunan Provincial People’s Hospital, The First Affiliated Hospital of Hunan Normal University, Changsha, 41005 China; 4 Department of Pediatric Surgery, Xinhua Hospital Affiliated to Shanghai Jiao Tong University School of Medicine, Shanghai, 200092 China; 5 Department of Hematology/Oncology, Guangzhou Women and Children’s Medical Center, National Children’s Medical Center for South Central Region, Guangzhou, 510623 China; 6 Department of Hematology, Shanghai Children’s Hospital, Shanghai Jiao Tong University, Shanghai, 200062 China; 7 Department of Pediatrics, West China Second University Hospital, Sichuan University, Chengdu, 610041 China; 8 Department of Hematology/Oncology, Qilu Children’s Hospital of Shandong University, Jinan, 250022 China; 9 Department of Pediatric Surgery, Children’s Hospital of Fudan University, Shanghai, 201102 China; 10 Department of General Surgery, Shanghai Children’s Medical Center (National Children’s Medical Center-Shanghai), Shanghai Jiao Tong University School of Medicine, Shanghai, 200127 China; 11 Department of Pediatrics, Beijing Tongren Hospital, Capital Medical University, Beijing, 100730 China; 12 Department of Pediatric Oncology, Tianjin Medical University Cancer Institute and Hospital, Tianjin, 300060 China; 13 Department of Hematology/Oncology, Children’s Hospital of Nanjing Medical University, Nanjing, 210008 China; 14 Department of Hematology/Oncology, Henan Children’s Hospital, Zhengzhou, 450018 China; 15 Department of Pediatric General Surgery, Hebei Children’s Hospital of Hebei Medical University, Shijiazhuang City, 050031 China; 16 Children’s Hospital, the First Affiliated Hospital of Zhengzhou University, Zhengzhou, 450052 China; 17 Department of Paediatric Hematology/Oncology, the Affiliated Hospital of Qingdao University, Qingdao, 266000 China; 18 Department of Paediatric Hematology/Oncology, Dalian Women and Children’s Medical Group, Dalian, 116037 China; 19 Department of Pediatric Hematology/Oncology, Shandong Provincial Hospital Affiliated to Shandong First Medical University, Jinan, 250021 China; 20 Department of General Surgery, Children’s Hospital of Soochow University, Suzhou, 215028 China; 21 Department of Pediatric Surgery, the Second Hospital of HeBei Medical University, Shijiazhuang, 050000 China; 22 Department of Paediatric Hematology/Oncology, the Third Affiliated Hospital of Zhengzhou University, Zhengzhou, 450052 China; 23 Department of Pediatric Surgery, the Second Affiliated Hospital and Yuying Children’s Hospital of Wenzhou Medical University, Wenzhou, 325027 China; 24 Department of Pediatrics, Xiangya Hospital Central South University, Changsha, 410008 China; 25 Department of Surgical Oncology, Beijing Children’s Hospital, Capital Medical University, National Center for Children’s Health, Beijing, 100045 China; 26 Clinical Research and Innovation Unit, XinHua Hospital Affiliated to Shanghai Jiao Tong University School of Medicine, Shanghai, 200092 China

**Keywords:** Alpha-fetoprotein, Hepatoblastoma, Multicenter, Prospective study, Survival

## Abstract

**Background:**

This study aimed to identify survival risk factors in Chinese children with hepatoblastoma (HB) and assess the effectiveness of the new treatment protocol proposed by the Chinese Children’s Cancer Group (CCCG) in 2016.

**Methods:**

A multicenter, prospective study that included 399 patients with HB from January 2015 to June 2020 was conducted. Patient demographics, treatment protocols, and other related information were collected. Cox regression models and Kaplan–Meier curve methods were used.

**Results:**

The 4-year event-free survival (EFS) and overall survival (OS) were 76.9 and 93.5%, respectively. The 4-year EFS rates for the very-low-risk, low-risk, intermediate-risk, and high-risk groups were 100%, 91.6%, 81.7%, and 51.0%, respectively. The 4-year OS was 100%, 97.3%, 94.4%, and 86.8%, respectively. Cox regression analysis found that age, tumor rupture (R +), and extrahepatic tumor extension (E +) were independent prognostic factors. A total of 299 patients had complete remission, and 19 relapsed. Patients with declining alpha-fetoprotein (AFP) > 75% after the first two cycles of neoadjuvant chemotherapy had a better EFS and OS than those ≤ 75%.

**Conclusions:**

The survival outcome of HB children has dramatically improved since the implementation of CCCG-HB-2016 therapy. Age ≥ 8 years, R + , and E + were independent risk factors for prognosis. Patients with a declining AFP > 75% after the first two cycles of neoadjuvant chemotherapy had better EFS and OS.

**Graphical abstract:**

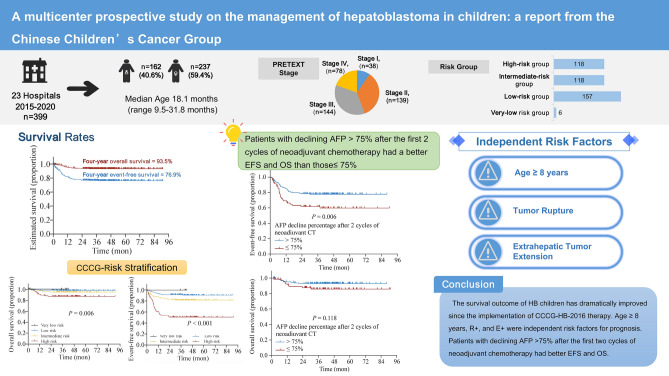

**Supplementary Information:**

The online version contains supplementary material available at 10.1007/s12519-023-00750-6.

## Introduction

Childhood and adolescent cancers are major public health concerns [1]. Hepatoblastoma (HB) is the most common hepatic malignancy in infants and children and accounts for about 50%–60% of primary malignant liver tumors. Over the past 30 years, with the rapid development of multidisciplinary treatment modalities and risk-stratification-based treatment strategies, the survival outcome of patients has dramatically improved, and the 5-year overall survival rate (OS) has reached 80%–90% [2]. Survival rates are better in patients with focal or resectable hepatoblastoma; however, poorer survival in patients with distant metastases or unresectable tumors is a current concern. In 2009, the Chinese Children’s Cancer Group (CCCG) developed the Treatment Protocol for Hepatoblastoma in Wuhan, China, commonly known as the Wuhan Protocol, which focused on preoperative chemotherapy and multidisciplinary treatment. Our team reported the feasibility and effectiveness of the Wuhan Protocol in 2016, in which the 6-year event-free survival (EFS) and OS were up to 71.0% and 83.3%, respectively [3].

Due to the low incidence of hepatoblastoma, several international research collaborative groups including International Childhood Liver Tumors Strategy Group (SIOPEL)/Gesellschaft fur Padiatrische Onkologie und Hamatologie (GPOH), Children's Oncology Group (COG), and Japanese Study Group for Pediatric Liver Tumor (JPLT) jointly established the Children’s Hepatic Tumors International Collaboration (CHIC) to identify novel prognostic factors and to establish a new stratification system in 2016 [4]. The CCCG proposed the Expert Consensus for Multidisciplinary Treatment of Hepatoblastoma (CCCG-HB-2016) in 2016 in China [5]. The protocol was revised and optimized in the following aspects: histopathologic examination criteria, pre-treatment extent of tumor (PRETEXT)/post-treatment extent of tumor (POSTTEXT) stage system, risk stratification system, surgical indications, and liver transplantation indications.

The protocol was extended to the whole country and implemented for 5 years. We herein report the results of patients treated with the CCCG-HB-2016 protocol of the CCCG and explore the potential prognostic factors of HB in Chinese children.

## Methods

### Patients and eligibility

This was a multicenter, prospective study that included 399 patients with HB from 23 hospitals from January 2015 to June 2020. The inclusion criteria were as follows: (1) age at diagnosis was < 18 years; (2) patient was chemotherapy-naïve; and (3) patients had received at least one cycle of CCCG-HB-2016 protocol treatment. The exclusion criteria of the study were as follows: (1) patients with severe liver and kidney dysfunction or cardiac dysfunction and (2) patients with other malignant tumors. The study was approved by the Ethics Committee of Xinhua Hospital affiliated to Shanghai Jiao Tong University School of Medicine, Shanghai, China, and informed consent was obtained from the patients’ guardians. The clinical registration number was ChiCTR1800017935.

### CCCG-HB-2016 protocol

All patients with HB were defined as very-low-risk, low-risk, intermediate-risk, and high-risk groups according to the CCCG-HB-2016 risk stratification system, based on the PRETEXT staging and COG staging [6, 7]. Except for the very-low-risk group, patients in other groups were treated with surgery and chemotherapy (neoadjuvant and postoperation chemotherapy) (Supplementary Fig. 1). The treatment plan for each risk group varied. The chemotherapy regimens for low-risk, intermediate-risk, and high-risk group patients were derived from a previously published reference (Supplementary Table 1) [5]. Chemotherapy was repeated every 3–4 weeks. Alpha-fetoprotein (AFP) level and B-scan ultrasonography were performed after every cycle. Magnetic resonance imaging (MRI) or computed tomography (CT) was performed after every two cycles of chemotherapy.

### Statistical analysis and evaluation of response

The last follow-up date was June 31, 2022, and the median follow-up was 40.5 months (ranging from 0.4 to 91.1 months). Complete remission (CR) means that there is no evidence of a tumor in CT or MRI and normal serum AFP levels for at least four weeks. Partial remission (PR) means a decrease of at least 50% in size of all measurable lesions, with no evidence of new lesions or progression in any lesion. Stable disease (SD) refers to any remission without an increase in tumor size or new lesions. Progressive disease (PD) refers to an increase of at least 25% in the size of any lesion, any new lesion, or a rising AFP level. EFS was calculated from the date of diagnosis to any event happening (including PD, recurrence, abandonment, or death, whichever occurred first). OS was calculated from the date of diagnosis to death. The patients who were lost to follow-up were censored at the last visit date.

Statistical analyses were performed with SPSS 24.0 (IBM, Chicago, IL, USA). Figures were plotted with GraphPad Prism 8.01 (GraphPad Software Inc., San Diego, CA, USA). Continuous variables were expressed as the median and interquartile range (IQR). Categorical variables are presented as frequencies (percentages). Comparison of AFP among different visit points was determined by Friedman’s rank test. The probabilities of EFS and OS were calculated by the Kaplan–Meier method and compared by the log-rank test. Univariable Cox proportional hazard regression analysis was performed to screen the factors predicting EFS and OS. Only the variables with *P* < 0.05 in univariable Cox proportional hazard analysis were further included in forward stepwise multivariable Cox proportional hazard regression models to assess the independent predictors of EFS and OS. All statistical tests were two-sided, and *P* < 0.05 was considered statistically significant.

## Results

### Clinical characteristics

A total of 399 patients with complete medical records were included in this study, including 162 (40.6%) males and 237 (59.4%) females, with a median age at diagnosis of 18.1 months (range: 9.5–31.8 months). In our study, 311 patients (77.9%) were < 3 years old, and 11 (2.8%) were > 7 years old. About half of the patients (200 cases, 50.1%) had an AFP level between 100,000 and 999,999 ng/mL at diagnosis (Table 1).

**Table 1:** Characteristics of 399 patients with hepatoblastoma

Characteristics	HB patients (*N* = 399)
Demographics	
Age (y), median (IQR)	18.1 (9.5–31.8)
< 3, *n* (%)	311 (77.9)
3–7, *n* (%)	77 (19.3)
≥ 8, *n* (%)	11 (2.8)
Gender, *n* (%)	
Female	237 (59.4)
Male	162 (40.6)
Birth weight (kg), median (IQR)	3.3 (3.0–3.6)
Very low birth weight infant, *n* (%)	12 (3.0)
Low birth weight infant, *n* (%)	13 (3.3)
Normal birth weight, *n* (%)	352 (88.2)
NA	22 (5.5)
Premature birth, *n* (%)	
No	360 (90.2)
Yes	35 (8.8)
NA	4 (1.0)
IVF, *n* (%)	
No	389 (97.5)
Yes	8 (2.0)
NA	2 (0.5)
Family history of HB, *n* (%)	
No	380 (95.2)
Yes	14 (3.5)
NA	5 (1.3)
Parental characteristics	
Mother’s age (y), median (IQR)	28.0 (25.0–32.0)
Father’s age (y), median (IQR)	30.0 (27.0–34.0)
Parent’s unhealthy lifestyle, *n* (%)	
No	357 (89.5)
Yes	40 (10.0)
NA	2 (0.5)
Risk factors during pregnancy, *n* (%)	
No	357 (89.5)
Yes	39 (9.8)
NA	3 (0.8)
Diagnosis methods, *n* (%)	
Biopsy	196 (49.1)
Surgery	116 (29.1)
Clinical diagnosis	87 (21.8)
Pathological subtypes, *n* (%)	
Pure fetal	98 (24.6)
Small cell undifferentiated (SCU)	4 (1.0)
Epithelial excluding pure fetal and SCU	125 (31.3)
Epithelial and mesenchymal	148 (37.1)
NA	24 (6.0)
Hepatitis virus infection, *n* (%)	
No	396 (99.2)
Yes	2 (0.5)
NA	1 (0.3)
Metastasis at the onset, *n* (%)	76 (19.0)
Pulmonary metastasis, *n* (%)	62 (15.5)
Bone metastasis, *n* (%)	8 (2.0)
Lymph-node metastasis, *n* (%)	11 (2.8)
Other metastases, *n* (%)	8 (2.0)
Tumor size (cm), median (IQR)	10.6 (9.0–12.6)
PRETEXT stage, *n* (%)	
I	38 (9.5)
II	139 (34.8)
III	144 (36.1)
IV	78 (19.6)
POSTTEXT stage, *n* (%)	
	283
I	73 (25.8)
II	125 (44.2)
III	49 (17.3)
IV	13 (4.6)
NA	23 (8.1)
COG Evans stage, *n* (%)	
I	250 (62.7)
II	24 (6.0)
III	49 (12.3)
IV	76 (19.0)
CCCG-HB-2016 risk stratification, *n* (%)	
Very-low-risk group	6 (1.5)
Low-risk group	157 (39.3)
Intermediate-risk group	118 (29.6)
High-risk group	118 (29.6)
CHIC risk stratification, *n* (%)	
Very low risk	66 (16.5)
Low risk	118 (29.6)
Intermediate risk	121 (30.3)
High risk	94 (23.6)
Annotation factors, *n* (%)	
P +	68 (17.0)
V +	76 (19.0)
E +	34 (8.5)
N +	38 (9.5)
R +	32 (8.0)
F +	79 (19.8)
AFP, *n* (%)	
< 100 ng/mL	3 (0.8)
100–999 ng/mL	28 (7.0)
1000–9999 ng/mL	62 (15.5)
10,000–99,999 ng/mL	80 (20.1)
100,000–999,999 ng/mL	200 (50.1)
≥ 1,000,000 ng/mL	26 (6.5)
Platelet (10^9^/L), median (IQR)	650.0 (508.0–833.0)
Thrombocytosis, *n* (%)	
No (< 450 × 10^9^/L)	31 (7.8)
Yes (≥ 450 × 10^9^/L)	186 (46.6)
NA	182 (45.6)
Treatment procedures	
Surgery, *n* (%)	
No	32 (8.0)
Yes	367 (92.0)
Surgery location, *n* (%) (*n* = 367)	
Irregularity	67 (18.3)
Right side	105 (28.6)
Left side	57 (15.5)
Both right and left sides	4 (1.1)
Others	103 (28.1)
NA	31 (8.4)
Timing of surgery, *n* (%)	
No. of assessed patients	367
Post-chemotherapy	251 (68.4)
Pre-chemotherapy	116 (31.6)
Treatment summary, *n* (%)	
Strictly follow consensus	295 (73.9)
Deviate consensus	89 (22.3)
NA	15 (3.8)
Liver transplantation, *n* (%)	4 (1.0)

*HB* hepatoblastoma, *IQR* interquartile range, *IVF* in-vitro fertilization, *PRETEXT* pre-treatment extent of tumor, *COG* Children’s Oncology Group, *CCCG-HB* Chinese Children’s Cancer Group of Hepatoblastoma, *CHIC* Children’s Hepatic tumors International Collaboration, *P*^+^ portal vein involvement, *V*^+^ inferior vena cava or hepatic vein involvement, *E*^+^ extrahepatic tumor extension, *N*^+^ lymph-node metastasis, *R*^+^ tumor rupture, *F*^+^ multifocal tumor, *AFP* alpha-fetoprotein, *POSTTEXT* post-treatment extent of tumor, *NA* not available

### Stage, pathology, and metastasis

Patients were classified by PRETEXT staging at diagnosis, POSTTEXT staging after neoadjuvant chemotherapy, and COG (Evans Surgical) staging after surgery. At diagnosis, 38 patients (9.5%) were in PRETEXT stage I, 139 (34.8%) in stage II, 144 (36.1%) in stage III, and 78 (19.6%) in stage IV. Among all the patients, 327 (82.0%) were positive for one of the PRETEXT annotation factors, including portal vein involvement (P +) (17.0%), inferior vena cava or hepatic vein involvement (V +) (19.0%), extrahepatic tumor extension (E +) (8.5%), and multifocal tumor (F +) (19.8%). After neoadjuvant chemotherapy, 47 patients (47/78, 60.2%) in PRETEXT stage IV were classified as POSTTEXT II (*n* = 27) and III (*n* = 21). Two hundred fifty patients (62.7%) were in COG stage I, 24 (6.0%) in stage II, 49 (12.3%) in stage III, and 76 (19.0%) in stage IV.

Half of the patients (227/399, 56.9%) were defined as epithelial subtype, the primary pathological subtypes in the study, which included 4 (1.0%) small cell undifferentiated (SCU) and 98 (24.6%) pure fetal type. One hundred forty-eight patients (37.1%) were defined as mixed epithelial and mesenchymal types, and 18 had no pathological subtypes. Six patients were diagnosed clinically and did not receive delayed operations.

Among the 399 patients, 62 (15.5%) had pulmonary metastasis, 8 (2.0%) had bone metastasis, and 11 (2.8%) had metastasis to the lymph nodes. Two patients (0.5%) had both lung and bone metastasis, and six patients (1.5%) had both lung and lymph-node metastasis.

### Treatment path and outcome

**Fig. 1:**
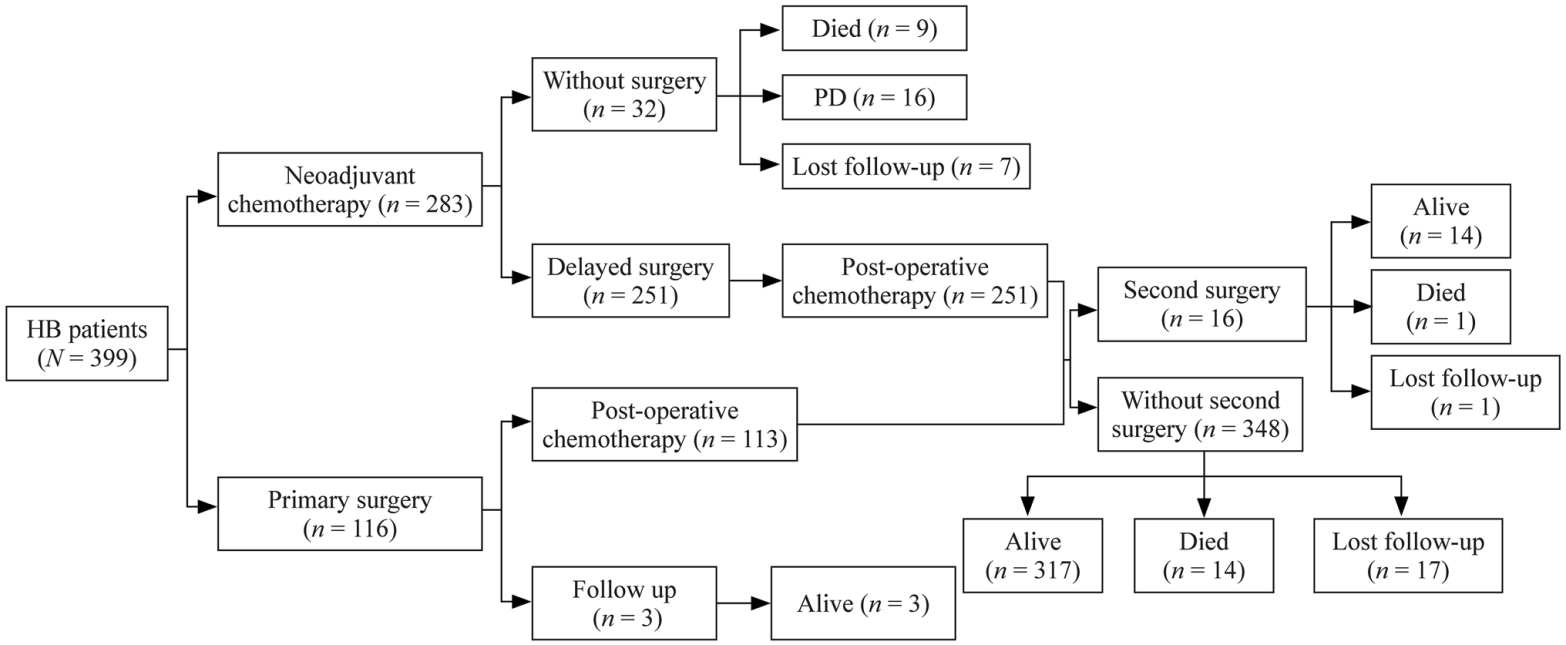
The treatment path and outcome of 399 HB patients. *HB* hepatoblastoma, *PD* progressive disease

The treatment path and outcome of the 399 HB patients are presented in Fig. 1. A total of 283 (70.9%) received neoadjuvant chemotherapy compared with 116 (29.1%) who underwent primary surgery. Among the 283 patients, 251 patients (88.7%) underwent delayed surgery after chemotherapy, and the remaining 32 (11.3%) did not undergo additional surgical resection. In total, 364 patients received postoperative chemotherapy. One hundred eleven patients suffered from at least one adverse event following chemotherapy, and the most common adverse event was septicemia (41 patients; 10.4%).

A total of 367 (367/399, 91.7%) patients underwent surgery, and complete tumor resection was performed in 333 (90.7%) patients. Of the patients who did not undergo surgery, 9 died, 16 had PD, and 7 were lost to follow-up. Among the 367 cases, the resection sites of 105 patients (28.6%) were in the right liver lobe, 57 (15.5%) in the left liver lobe, and 4 (1.1%) in both right and left. The resection site of 67 patients (18.3%) was irregular, and the remaining cases were unknown. After surgery, adverse events occurred in 10 patients, with ascites (6 patients; 1.6%) being the most prevalent side effect (Supplementary Table 2). Twelve patients (3.0%) underwent a second surgery, including one for postoperative complications, four for pulmonary metastasectomy, and seven for recurrent intrahepatic tumor resection.

Thirty-nine patients who did not achieve complete remission after first-line treatment (chemotherapy combined with surgery) were treated with a “second-line therapy,” which consisted of transarterial chemoembolization (TACE) (*n* = 31), radiofrequency ablation (RFA) (*n* = 3), and high-intensity focused ultrasound (HIFU) (*n* = 5).

At the end of the follow-up, 299 (299/399, 74.9%) patients had complete remission, 24 (6.0%) patients died, 17 for primary disease, 5 for relapse, and 2 for adverse effects. Twenty patients had relapses, 4 for pulmonary relapse, and 17 for liver relapse. Sixteen (4.0%) patients had PD, and 25 patients (6.27%) were lost to follow-up.

### Survival and prognostic factors

**Fig. 2:**
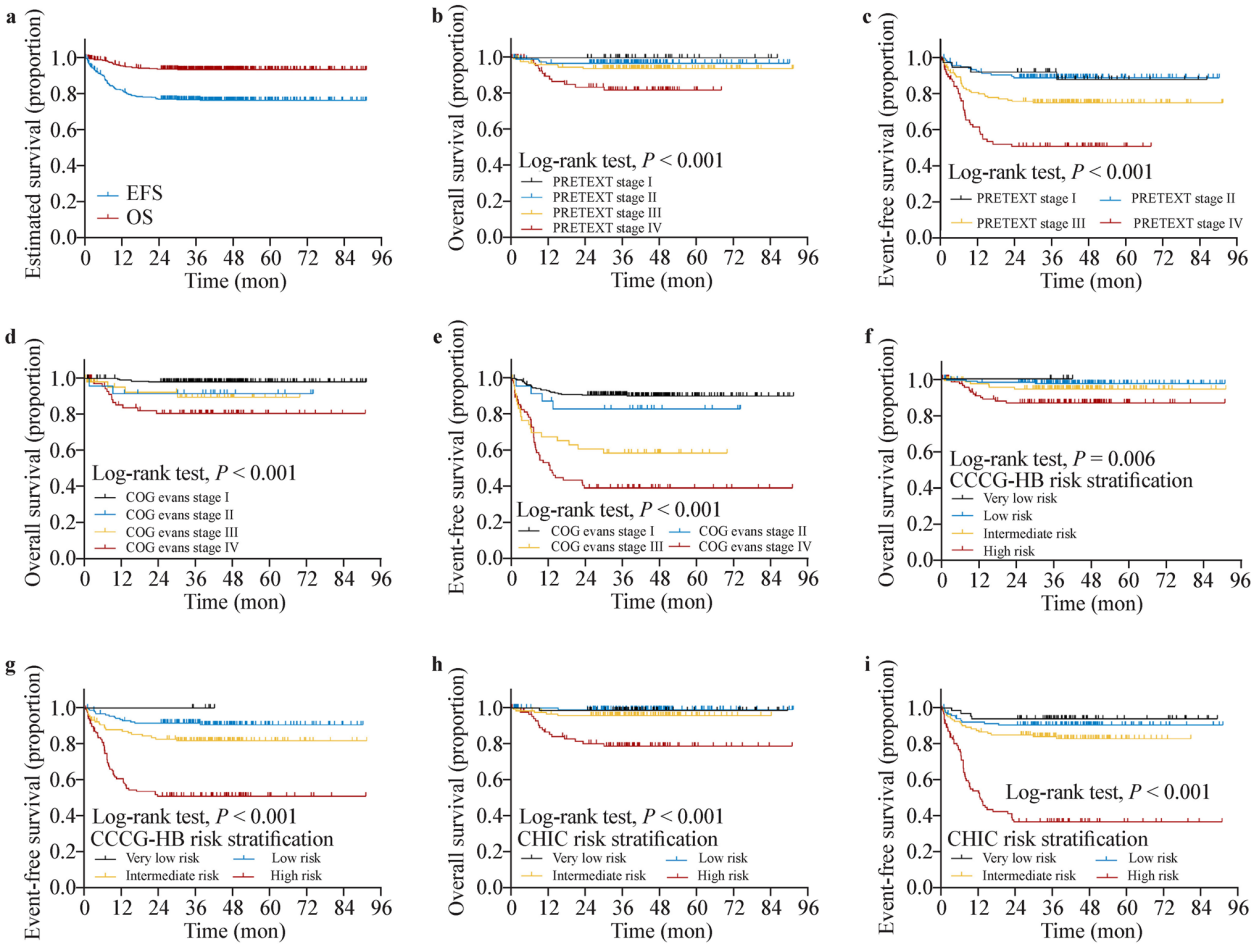
The event-free survival (EFS) and overall survival (OS) of 399 HB patients treated with CCCG-HB-2016 protocol under different risk stratification system. **a** The EFS and OS for all the 399 HB patients. **b**, **c** The OS and EFS of 399 HB patients with different PRETEXT stages. **d**, **e** The OS and EFS of 399 HB patients with different COG Evans stages. **f**, **g** The OS and EFS of 399 HB patients with different CCCG-HB-2016 risk stratification. **h**, **i** The OS and EFS of 399 HB patients with different CHIC risk stratification. *CCCG *Chinese Children’s Cancer Group,* HB* hepatoblastoma, *PRETEXT* pre-treatment extent of tumor,* COG* Children's Oncology Group,* CHIC* Children’s Hepatic Tumors International Collaboration

The 4-year EFS was 76.9% [95% confidence interval (CI) 72.8–81.2%], and the 4-year OS was 93.5% (95% CI 91.0–96.1%). The 4-year EFS for patients in the very-low-risk group, low-risk group, intermediate-risk group, and high-risk group was 100.0%, 91.6% (87.3–96.1%), 81.7% (75.0–89.1%), and 51.0% (42.6–61.1%), respectively. The 4-year OS for the corresponding risk groups were 100.0%, 97.3% (94.7–100.0%), 94.4% (90.1–98.9%), and 86.8% (80.6–93.5%), respectively. The survival rates for PRETEXT, COG stage, and CHIC and risk group are detailed in Fig. 2 and Table 2.

**Table 2:** Comparison of the EFS and OS in patients with hepatoblastoma based on different stratification

Variables	No	EFS			OS		
		4-year EFS (95% CI)	HR (95% CI)	*P* value	4-year OS (95% CI)	HR (95% CI)	*P* value
Total	399	76.9% (72.8–81.2%)			93.5% (91.0–96.1%)		
Age, y							
< 3	311	82.0% (77.7–86.6%)	1	Reference	95.3% (92.8–97.8%)	1	Reference
3–7	77	66.7% (57.8–77.0%)	1.994 (1.278–3.112)	**0.002**	87.0% (80.1–94.5%)	2.725 (1.221–6.084)	**0.014**
≥ 8	11	34.2% (15.5–75.2%)	4.941 (2.344–10.417)	** < 0.001**	100.0%	–	**–**
Gender							
Female	237	77.9% (72.7–83.4%)	1	Reference	93.7% (90.6–97.0%)	1	Reference
Male	162	75.4% (69.0–82.4%)	1.096 (0.723–1.659)	0.667	93.2% (89.3–97.4%)	1.046(0.465–2.355)	0.913
Serum AFP level at diagnosis, ng/mL							
<100,000	172	86.5% (81.5–91.8%)	1	Reference	98.2% (96.2–100.0%)	1	Reference
>100,000	227	69.4% (63.5–75.8%)	2.393 (1.501–3.816)	** < 0.001**	89.7% (85.6–94.0%)	5.954(1.776–19.965)	**0.004**
Pathological subtypes							
Fetal	89	81.7% (74.0–90.2%)	1	Reference	91.6% (85.9–97.8%)	1	Reference
Embryonal	27	84.5% (71.7–99.7%)	0.891 (0.298–2.664)	0.836	92.3% (82.5–100.0%)	0.982(0.204–4.728)	0.982
SCU	5	60.0% (29.3–100.0%)	2.681 (0.616–11.666)	0.189	80.0% (51.6–100.0%)	2.821(0.347–22.932)	0.332
Mixed	270	76.2% (71.3–81.5%)	1.370 (0.792–2.369)	0.261	95.2% (92.6–97.9%)	0.569(0.224–1.444)	0.235
PRETEXT stage							
I	38	92.1% (83.9–100.0%)	1	Reference	100.0%	1	Reference
II	139	89.0% (83.9–94.4%)			97.0% (94.2–99.9%)		
III	144	75.0% (68.1–82.5%)	2.515 (1.438–4.396)	**0.001**	94.0% (90.0–98.1%)	2.649(0.798–8.796)	0.112
IV	78	50.8% (40.7–63.5%)	5.787 (3.324–10.074)	** < 0.001**	82.1% (73.3–91.8%)	7.951(2.563–24.663)	** < 0.001**
PRETEXT annotation factors (VPEFR)							
P +	76	62.2% (52.0–74.3%)	2.204 (1.418–3.426)	** < 0.001**	88.8% (81.7–96.4%)	2.154(0.922–5.033)	0.076
P −	323	80.3% (76.1–84.9%)	1	Reference	94.6% (92.1–97.2%)	1	Reference
V +	68	65.3% (54.8–77.8%)	1.918 (1.195–3.078)	**0.007**	85.1% (76.5–94.6%)	3.171(1.388–7.247)	**0.007**
V −	331	79.3% (75.0–83.8%)	1	Reference	95.2% (92.8–97.6%)	1	Reference
E +	34	37.7% (24.2–58.9%)	4.076 (2.478–6.705)	** < 0.001**	72.4% (57.8–90.7%)	6.534(2.794–15.277)	** < 0.001**
E −	365	80.4% (76.4–84.6%)	1	Reference	95.3% (93.1–97.6%)	1	Reference
F +	79	54.4% (44.5–66.6%)	3.045 (1.999–4.639)	** < 0.001**	86.2% (78.7–94.5%)	3.001(1.333–6.759)	**0.008**
F −	320	82.6% (78.5–86.9%)	1	Reference	95.3% (92.9–97.7%)	1	Reference
R +	32	58.1% (43.1–78.4%)	2.045 (1.137–3.679)	**0.017**	79.8% (66.5–95.7%)	4.219(1.674–10.632)	**0.002**
R −	367	78.6% (74.4–82.9%)	1	Reference	94.7% (92.3–97.1%)	1	Reference
Lymph-node infiltration							
Positive	38	77.7% (64.3–93.9%)	4.743 (1.965–11.450)	** < 0.001**	79.4% (66.9–94.2%)	4.445(1.843–10.720)	** < 0.001**
Negative	361	94.6% (92.2–97.2%)	1	Reference	94.9% (92.6–97.3%)	1	Reference
POSTTEXT stage							
I	73	80.4% (71.6–90.2%)	1	Reference	97.1% (93.3–100.0%)	1	Reference
II	125	81.5% (74.9–88.6%)	0.905 (0.472–1.734)	0.763	96.6% (93.4–99.9%)	1.195(0.219–6.522)	0.837
III	49	64.0% (51.7–79.3%)	1.948 (0.972–3.901)	0.060	88.7% (79.9–98.5%)	4.083(0.792–21.047)	0.093
IV	13	7.7% (1.17–50.6%)	8.107 (3.761–17.477)	** < 0.001**	47.6% (23.0–98.5%)	18.698(3.616–96.695)	** < 0.001**
COG Evans stage							
I	250	90.7% (87.2–94.4%)	1	Reference	97.9% (96.2–99.7%)	1	Reference
II	24	83.1% (69.3–99.7%)	1.802 (0.625–5.193)	0.276	91.7% (81.3–100.0%)	4.356(0.845–22.455)	0.079
III	49	58.5% (45.8–74.8%)	5.560 (3.044–10.156)	** < 0.001**	89.5% (80.2–99.9%)	5.444(1.461–20.281)	**0.012**
IV	76	39.3% (29.4–52.3%)	8.888 (5.391–14.656)	** < 0.001**	80.5% (71.6–90.6%)	10.539(3.755–29.581)	** < 0.001**
Metastasis at the onset							
Yes	76	39.3% (29.4–52.3%)	5.584 (3.691–8.448)	** < 0.001**	80.5% (71.6–90.6%)	5.930(2.655–13.246)	** < 0.001**
No	323	85.5% (81.7–89.4%)	1		96.4% (94.3–98.5%)	1	
Lung	62	35.0% (24.6–49.7%)	5.747 (3.776–8.747)	** < 0.001**	82.9% (73.3–93.8%)	3.871(1.693–8.849)	**0.001**
Lymph nodes	11	54.5% (31.8–93.6%)	2.701 (1.096–6.658)	**0.031**	88.9% (70.6–100.0%)	1.875(0.253–13.888)	0.538
CCCG -HB -2016 risk group							
Very-low-risk group	6	100.0%	1	Reference	100.0%	1	Reference
Low-risk group	157	91.6% (87.3–96.1%)			97.3% (94.7–100.0%)		
Intermediate-risk group	118	81.7% (75.0–89.1%)	2.233 (1.136–4.392)	**0.020**	94.4% (90.1–98.9%)	2.276(0.642–8.066)	0.207
High-risk group	118	51.0% (42.6–61.1%)	7.360 (4.093–13.179)	** < 0.001**	86.8% (80.6–93.5%)	5.621(1.850–17.084)	**0.002**
CHIC risk group							
Very-low-risk group	66	93.9% (88.4–99.9%)	1	Reference	98.5% (95.6–100.0%)	1	Reference
Low-risk group	118	90.5% (85.2–96.0%)	1.635 (0.520–5.133)	0.400	92.2% (97.3–100.0%)	0.604(0.038–9.651)	0.721
Intermediate-risk group	121	84.1% (77.8–90.9%)	2.935 (1.003–8.586)	**0.049**	95.7% (92.1–99.5%)	2.928(0.342–25.061)	0.327
High-risk group	94	36.7% (27.9–48.3%)	15.144 (5.482–41.829)	** < 0.001**	78.8% (70.3–88.3%)	15.210(2.023–114.340)	**0.008**
Complications after chemotherapy							
Yes	111	70.8% (62.6–80.0%)	1.460 (0.946–2.252)	0.874	72.8% (64.6–82.0%)	1.678(1.084–2.597)	**0.020**
No	285	78.9% (74.3–83.9%)	1	Reference	87.6% (83.7–91.7%)	1	Reference
Tumor size							
≥ 10 cm	259	72.2% (66.8–77.9%)	1.947 (1.195–3.170)	**0.007**	80.4% (75.5–85.7%)	1.894(1.162–3.086)	**0.010**
< 10 cm	138	85.3% (79.5–91.4%)	1	Reference	89.3% (84.2–94.8%)	1	Reference

*EFS* event-free survival, *OS* overall survival, *HR* hazard ratio, *CI* confidence interval, *P* + portal vein involvement, *V* + inferior vena cava or hepatic vein involvement, *E* + extrahepatic tumor extension, *N* + lymph-node metastasis, *R* + tumor rupture, *F* + multifocal tumor, *PRETEXT* pre-treatment extent of tumor, *COG* Children’s Oncology Group, *CCCG-HB* Chinese Children’s Cancer Group of Hepatoblastoma, *CHIC* Children’s Hepatic tumors International Collaboration, *SCU* small cell undifferentiated, *POSTTEXT* post-treatment extent of tumor

*P* < 0.05 are shown in bold characters

The prognostic factors, including age, AFP level at diagnosis, pathological subtypes, PRETEXT stage, PRETEXT annotation factors (VPEFR), POSTTEXT stage, COG stage, and metastasis at onset, were analyzed in our study. The survival rates and hazard ratios are shown in Table 2.

Multivariable cox regression analysis revealed that compared with age [3–7 years vs. < 3 years, hazard ratio (HR): 5.428, 95% CI 1.523–19.345; *P* = 0.009], E + (HR: 3.975, 95% CI 1.033–15.286; *P* = 0.045) and tumor rupture (R +) (HR: 7.044, 95% CI 1.784–27.806; *P* = 0.005) were independent risk factors for OS (Supplementary Table 3).

### Second-line treatment for patients (hepatoblastoma)

The propensity score matching (PSM) method was used to explore the impact of second-line therapies on survival. This was calculated by a logistic regression model with the following covariates: age, sex, and CCCG-HB 2016 risk stratification. The matching was performed using a 1:4 nearest-neighbor matching protocol with a caliper width of 0.2. The results showed that the 4-year EFS and OS of patients (*n* = 34) with second-line therapy were lower than those of patients who did not receive additional treatment (*n* = 129). However, this difference was not statistically significant (4-year EFS, *P* = 0.072; 4-year OS, *P* = 0.255) (Supplementary Fig. 2).

### Correlation between declining alpha-fetoprotein with event-free survival and overall survival rate

**Fig. 3:**
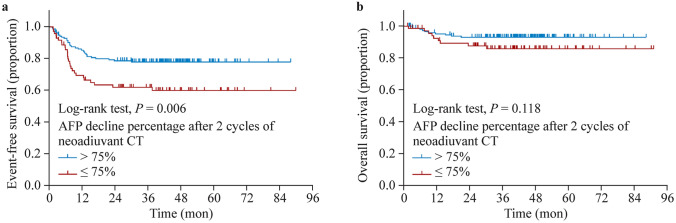
Correlation of AFP decline percentage with EFS and OS (*n* = 197). *AFP* alpha-fetoprotein, *EFS* event-free survival,* OS* overall survival,* CT* chemotherapy

This study found that the AFP level of HB patients continued to decline with the progression of treatment (Supplementary Fig. 3). By analyzing the relationship between the declining AFP during different treatment periods and patient prognosis, we found that the declining percentage of AFP after the first two cycles of neoadjuvant chemotherapy was positively correlated with EFS. Therefore, we further explored the relationship between AFP and EFS and established that AFP decline > 75% was statistically associated with EFS (Table 3). In addition, a stratified analysis with a 75% decrease in AFP as the cut-off value showed that patients with an AFP decline > 75% after the first two cycles of neoadjuvant chemotherapy had better EFS and OS than those with ≤ 75% (Fig. 3).

**Table 3:** Stratification analysis of the association between AFP decline percentage after two cycles of neoadjuvant chemotherapy and EFS

Items	Univariable Cox’s regression model			
	*P* value	HR	95% CI	
			Lower	Higher
AFP decline percentage after two cycles of neoadjuvant CT				
≤ 25%	Reference	–	–	–
25–50%	0.086	0.335	0.096	1.166
50–75%	0.920	0.959	0.426	2.161
> 75%	**0.004**	0.399	0.213	0.747

*AFP* alpha-fetoprotein, *EFS* event-free survival, *HR* hazard ratio, *CI* confidence interval

AFP decline percentage was calculated using the formula as follows: AFP decline percentage = AFP level at different timepoint-baseline AFP level/baseline AFP level × 100%; *P* < 0.05 are shown in bold characters

## Discussion

In 2016, our team first reported China's national survival rates for hepatoblastoma. After the protocol was revised based on the study results, the CCCG Collaborative Group adopted the CCCG-HB-2016 protocol from 2016 and used it in more collaborating hospitals. Compared with the 2016 reports, the number of participating collaborators increased from the original 13 hospitals to 23 hospitals. Our study also demonstrated an increased OS (93.5 vs. 83.3%) and EFS (76.9 vs. 71.0%) of hepatoblastoma patients compared with the CCCG-HB-2009 protocol. Very-low-risk, low-risk, and intermediate-risk group patients obtained satisfactory results in our study compared with other study groups [8–10].

However, the high-risk group patients still had poor survival rates, especially EFS rates. In the latest SIOPEL study for high-risk patients, the 3-year EFS and OS were 76 and 83%, respectively [11]. There were several potential reasons for the lower EFS rates in Chinese patients; the first and most important reason was the lower rates of liver transplantation. In the SIOPEL-4 study, half of the patients (8/16) with initial PRETEXT IV tumors underwent liver transplantation (LT); in contrast, in our study, only three patients underwent LT. LT can effectively improve the survival rates of patients with unresectable tumors, and the 5-year OS of HB patients receiving LT was 75.1% [12]. The lack of a source of liver donation and the high cost of treatment are current barriers to the spread of liver transplantation in children. In our country, the source of LT relies on living transplantation because of the absence of social and cultural acceptance of cadaveric donation.

In our study, we also further explored the risk factors in the high-risk group. As we know, initial PRETEXT IV tumor, PRETEXT annotation factors positive (P + , V +), and metastases were the risk classification criteria in the CCCG-HB-2016 protocol. The 4-year EFS and OS for PRETEXT IV tumors were 50.87% and 82.1%, 62.2% and 88.8% for P-positive tumors, 65.3% and 85.1% for V-positive tumors, and 39.3% and 80.5% for metastatic patients. The previous CHIC study demonstrated that the EFS of PRETEXT IV tumors was 60% [13], which was higher than ours, whereas the EFS of P-positive and V-positive tumors were 49% and 51% [4], respectively, which were lower than our study. The results above showed that P- and V-positive patients had a particular effect on the existing protocol, while PRETEXT IV and metastatic patients still had a poor effect. The main reason for the poor prognosis is that the tumor cannot be completely resectable after regular chemotherapy.

Although there were many difficulties for those unresectable tumors, we also tried to explore other treatments, including TACE, HIFU, and RFA, also called second-line therapy. Our study further analyzed the situation of patients who received the second-line treatment, and half of them were in the high-risk group (39.5%, 15/38). Second-line therapy led to a better survival outcome in patients compared to those who did not receive further treatment. However, this finding was not statistically significant. It is still controversial whether the addition of TACE, RFA, and HIFU treatment is beneficial to patients who responded poorly to chemotherapy combined with surgery [14–16]. The second-line therapy may help patients who do not want to undergo liver transplantation achieve tumor resection. The effectiveness and safety should be validated in future studies with a larger sample with a long-term follow-up.

Patients with initial metastasis have been another problem in treatment. Those patients have no indication for liver transplantation and have a worse prognosis and a higher mortality rate [17]. To date, the 3- to 5-year EFS rates have ranged from 20% to 76% [11, 18–20], and the best outcomes for patients with metastasis resulted from the SIOPEL-4 study. Although significant toxicity was also noted in SIOPEL-4, 97% of patients had grade 3–4 hematological toxicity. In the CCCG-HB-2016 protocol, the same therapeutic regimen of cisplatin combined with adriamycin was applied to metastatic patients, considering the higher prognosis of SIOPEL-4. However, compared with SIOEPL-4, the 4-year EFS was much lower (51.0% vs. SIOPEL-4: 76%) and higher than the CHIC results (42%). The reasons for the failures of metastatic patients were considered the adverse effects of chemotherapy and no response to treatment. Reducing the adverse effects of chemotherapy, improving the efficacy of chemotherapy, and standardizing the surgical procedures for lung metastasectomy need further focus and optimization in future protocols. Patients with initial metastasis and its impact on their survival rate have been a concern and focus in further studies.

In this study, prognostic factors reported in the previous studies were analyzed, including PRETEXT staging, PRETEXT annotation factors (VPEFR), and AFP levels; and the findings were consistent with previous data. We are also looking for new prognostic factors. Recent studies have shown that age has been added to further risk stratification criteria [21, 22]. We also analyzed the age of children in China and found a worse survival and higher hazard ratio for children aged over 8 years. We will add the age to the new stratification criteria in the future protocol. We also  analyzed the relationship between decreasing percentage AFP levels and survival rates. We found that patients whose AFP declined > 75% (after two cycles of neoadjuvant chemotherapy) had significantly better EFS and OS than those ≤ 75%. This finding suggested that the decline in AFP could be used as an indication of early treatment efficacy. Clinicians can assess chemotherapy efficacy through early AFP changes. However, this hypothesis should be validated with large-scale population studies. Moreover, adjusting the chemotherapy regimen according to AFP changes may be feasible. Patients with a declining AFP > 75% should reduce the intensity of chemotherapy to reduce adverse effects. Patients with an AFP decline ≤ 75% can have long-term outcome improved by following treatment regimens that refer to higher-risk patients.

Complications after chemotherapy were confirmed to be a risk factor for OS. The most common complications in our study included septicemia, lower respiratory tract infection, and disturbance of electrolyte balance. Septicemia, infection, and electrolyte disorder were independent risk factors for death in hospitalized patients [23]. Patients with adverse events would have a lower OS. However, patient support care may vary, as well as the center's experience with chemotherapy. These factors can influence patient care, which may lead to complications.

In conclusion, the survival outcome of children with HB has gradually improved since the implementation of the CCCG-HB-2016 protocol. Age, PRETEXT stage, and PRETEXT annotation factors (E + , R +) were independent prognostic factors. Patients with serum AFP decline > 75% (after two cycles of neoadjuvant chemotherapy) had better EFS and OS than those ≤ 75%, which suggested that the decline of AFP could be used as an indication of early treatment efficacy.

### Supplementary Information

Below is the link to the electronic supplementary material.

Supplementary file1 (DOCX 904 KB)

## Data Availability

The datasets during and/or analyzed during the current study are available from the corresponding author upon reasonable request.
