# Supplementary material for: A multicenter prospective study on the management of hepatoblastoma in children: a report from the Chinese Children’s Cancer Group
Source: World J Pediatr. 2023 Sep 28;20(6):590–601. doi: 10.1007/s12519-023-00750-6 (PMC11239770; doi:10.1007/s12519-023-00750-6)
Supplement: Supplementary file 1 — Supplementary file1 (DOCX 904 KB) [file 12519_2023_750_MOESM1_ESM.docx]

**Table S1.** Chemotherapy regimen of the CCCG-HB-2016 protocol

| Risk group | Chemotherapy regimen | Total course |
| --- | --- | --- |
| very-low risk | —— |  |
| low-risk | C5V(cisplatin, 5-FU,vincristine) :cisplatin (90 mg/m2) on day 1, 5- fluorouracil (600 mg/m2) and vincristine (1.5 mg/m2) on day 2. Treatment cycles were repeated every 21 days. | four to six |
| intermediate-risk | C5VD (cisplatin,5-FU, vincristine, doxorubicin) chemotherapy regimen was used for a total of six to eight courses. Which consisted of cisplatin (90 mg/m2) on day 1, 5- fluorouracil (600 mg/m2) and vincristine (1.5 mg/m2) on day 2, doxorubicin(25 mg/m2) on day 2 and day3. After two to four courses of chemotherapy, the surgical resection could be performed. Treatment cycles were repeated every 21-28 days. | six to eight |
| high-risk | C-CD (cisplatin, doxorubicin) + ICE (Ifosfamide, Carboplatin, Etoposide(VP16)) ：The C-CD regimen was performed for three cycles on pre-operation: First cycle: cisplatin (80 mg/m2) on day1, cisplatin (70 mg/m2) on day8, doxorubicin (30 mg/m2) on day8 and day9; Second cycle: cisplatin (70 mg/m2) on day1and day8 , doxorubicin (30 mg/m2) on day8 and day9; Third cycle: consistent with the second cycle. When patients had finished the pre-operation chemotherapy and able to undergo surgery, surgery will be performed. And patients will receive three post-operation consolidation courses. The post-operation chemotherapy regimen was consisted of Carboplatin (500 mg/m2) on day1, doxorubicin (20 mg/m2) on day1and day2. If patients had finished three cycles of pre-operative chemotherapy, but can’t undergo surgery, the ICE regimen would be used for another two cycles. Ifosfamide (1.5 mg/m2) on days 1 through 4, Carboplatin (450 mg/m2) on day 1, VP16 (100mg/m2) on days 1 through 4. After three to five courses of chemotherapy regimen, the surgical resection could be performed. After restriction, the ICE regimen will be performed for two cycles. Treatment cycles were repeated every 28 days. The ICE regimen was repeated for two cycles post-operation. | six to seven |

**Table S2. Adverse events after surgery and chemotherapy**

| Items | No. (%) |
| --- | --- |
| **Complication after surgery** | 10 (2.5) |
| Ascites | 6 (1.6) |
| Intestinal obstruction | 3 (0.8) |
| Infection | 3 (0.8) |
| Biliary fistula | 3 (0.8) |
| Liver injury | 1 (0.3) |
| Brain edema | 1 (0.3) |
| **Complication after chemotherapy** | 111 (28.0) |
| Septicemia | 41 (10.4) |
| Lower respiratory tract infection | 34 (8.6) |
| Upper respiratory tract infection | 14 (3.5) |
| Infection of digestive canal | 12 (3.0) |
| Other infections | 17 (4.3) |
| Electrolyte disturbance | 30 (7.6) |
| Renal dysfunction | 10 (2.5) |
| Hypoproteinemia | 7 (1.8) |
| Convulsion | 5 (1.3) |
| Coagulation disorders | 5 (1.3) |
| Liver function damage | 3 (0.8) |
| Others | 7 (1.8) |

**Table S3. Forward stepwise multivariate Cox’s hazard regression model analysis of risk factors independently predicting EFS and OS**

| Items | Multivariate Cox’s regression model | | | |
| --- | --- | --- | --- | --- |
|  | *P* value | HR | 95%CI | |
|  |  |  | Lower | Higher |
| **EFS** |  |  |  |  |
| Age |  |  |  |  |
| <3 years | Reference | - | - | - |
| 3-7 years | 0.019 | 1.969 | 1.118 | 3.468 |
| ≥8 years | 0.101 | 2.493 | 0.836 | 7.435 |
| COG | 0.005 | 1.745 | 1.185 | 2.569 |
| CCCG -HB -2016 Risk Group | 0.027 | 1.721 | 1.064 | 2.786 |
| **OS** |  |  |  |  |
| Age |  |  |  |  |
| <3 years | Reference | - | - | - |
| 3-7 years | 0.009 | 5.428 | 1.523 | 19.345 |
| ≥8 years | 0.998 | - | - | - |
| R+ | 0.005 | 7.044 | 1.784 | 27.806 |
| E+ | 0.045 | 3.975 | 1.033 | 15.286 |

EFS, event-free survival; OS, overall survival; HR, hazard ratio; CI, confidence interval; PRETEXT, pre-treatment extent of disease; COG, Children’s Oncology Group; R^+^, tumor rupture; E+, extrahepatic tumor extension

**
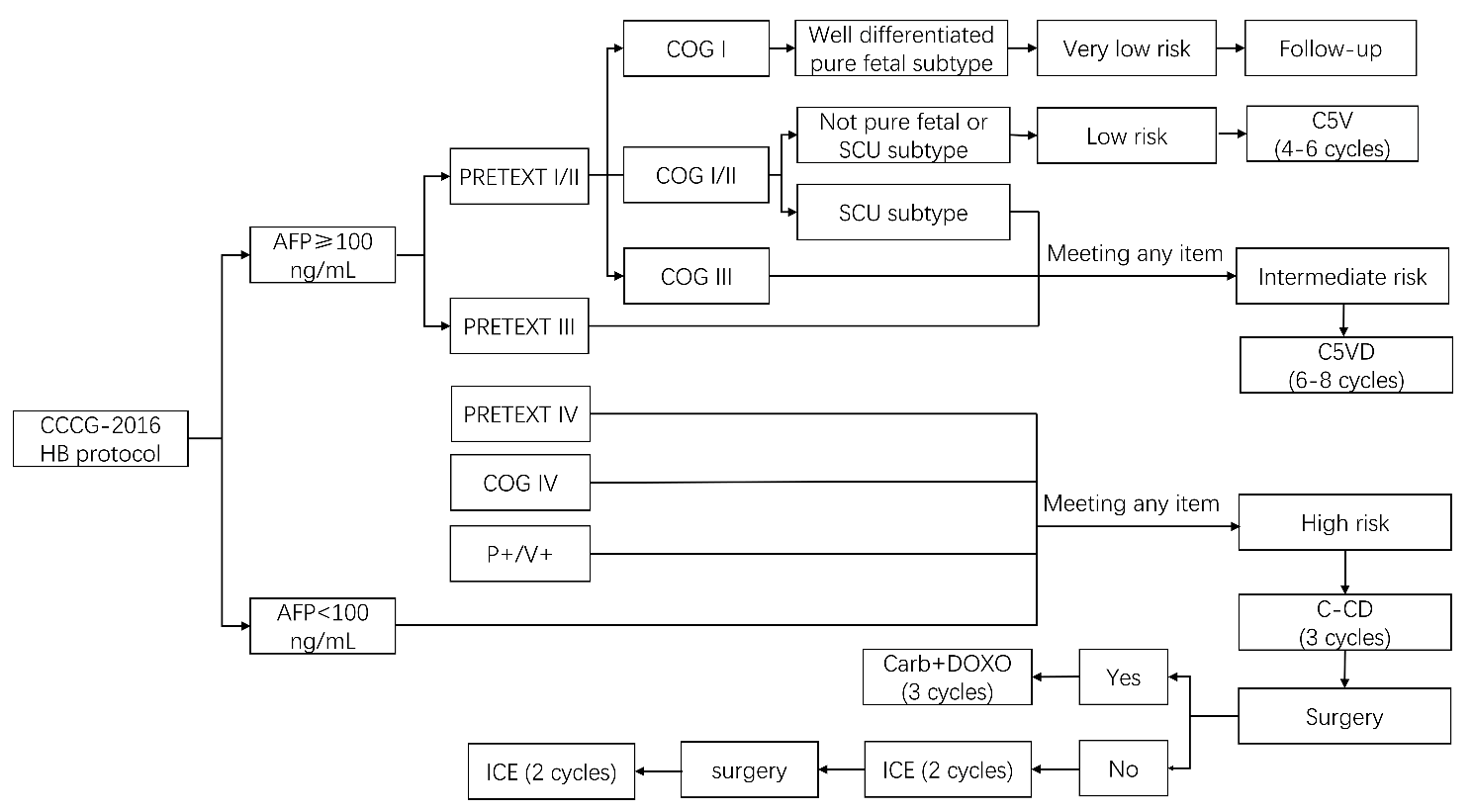
**

**Figure S1. Treatment protocol of CCCG-HB-2016**

C5V=cisplatin, fluorouracil and vincristine; C5VD=cisplatin, fluorouracil, vincristine and doxorubicin; C-CD=cisplatin and doxorubicin; ICE= ifosfamide, carbo and etoposide; Carb=Carbo, DOXO=doxorubicin


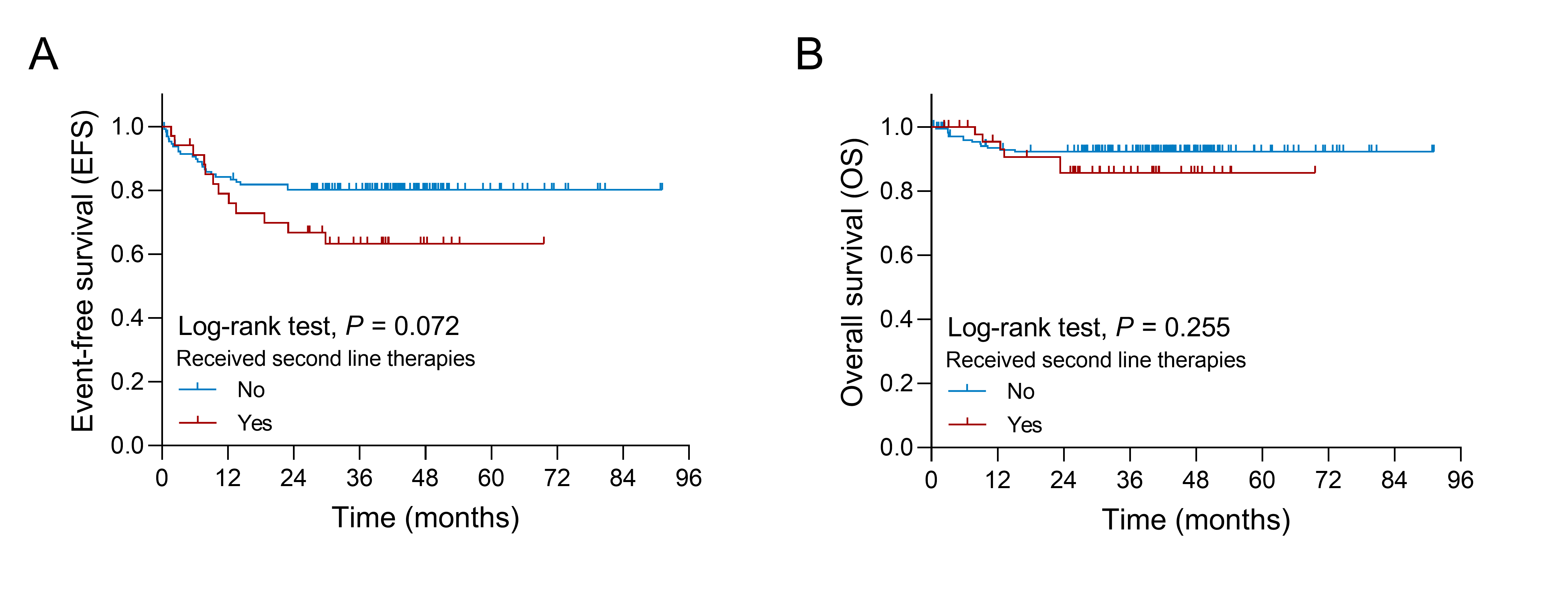


**Figure S2. The EFS and OS of HB patients received second-line therapy**


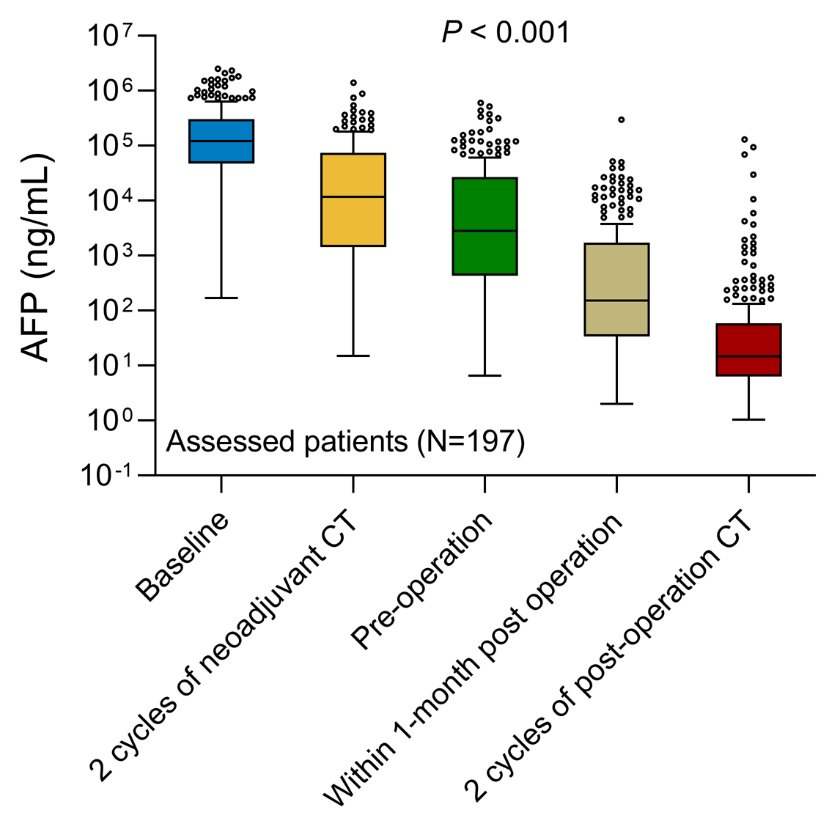


**Figure S3. Varity of AFP during the treatment period(n=197)**
